# Masters of manipulation: Viral modulation of the immunological synapse

**DOI:** 10.1111/cmi.12944

**Published:** 2018-09-21

**Authors:** Rebecca J. Bayliss, Vincent Piguet

**Affiliations:** ^1^ Division of Infection and Immunity, School of Medicine Cardiff University Cardiff UK; ^2^ Division of Dermatology, Department of Medicine University of Toronto Toronto Ontario Canada; ^3^ Division of Dermatology Women's College Hospital Toronto Ontario Canada

**Keywords:** dendritic cell, HIV‐1, HTLV‐1, immunological synapse, T‐cell, virological synapse, virus

## Abstract

In order to thrive, viruses have evolved to manipulate host cell machinery for their own benefit. One major obstacle faced by pathogens is the immunological synapse. To enable efficient replication and latency in immune cells, viruses have developed a range of strategies to manipulate cellular processes involved in immunological synapse formation to evade immune detection and control T‐cell activation.

In vitro, viruses such as human immunodeficiency virus 1 and human T‐lymphotropic virus type 1 utilise structures known as virological synapses to aid transmission of viral particles from cell to cell in a process termed *trans*‐infection. The formation of the virological synapse provides a gateway for virus to be transferred between cells avoiding the extracellular space, preventing antibody neutralisation or recognition by complement.

This review looks at how viruses are able to subvert intracellular signalling to modulate immune function to their advantage and explores the role synapse formation has in viral persistence and cell‐to‐cell transmission.

## 1 INTRODUCTION

The adaptive immune response is essential for the control of pathogen invasion and is regulated by co‐ordinated communication between immune cells. This contact is directed either via membrane‐bound receptors or via the secretion of cytokines and lytic granules in response to chemokines on the surface of antigen‐presenting cells (APCs). The interaction has been termed the immunological synapse (IS), a specialised zone of contact between two immune cells to allow the exchange of materials. Synapses can be formed between two cells, T‐cell–T‐cell (Dustin et al., 1998; Monks, Freiberg, Kupfer, Sciaky, & Kupfer, 1998), T‐cell–B‐cell (Batista, Iber, & Neuberger, 2001), and APC–T‐cell (Grakoui et al., 1999); however, the majority of work has centred on the latter. Numerous viruses including human immunodeficiency virus (HIV), respiratory syncytial virus, and herpesvirus have evolved to express viral proteins that specifically target components of the IS with particular emphasis on the T‐cell receptor (TCR) signalling cascade and lymphocyte function‐associated antigen 1 (LFA‐1) clustering, both essential for IS formation.

Classically, viruses initiate contact with a host cell via attachment to a specific receptor on the target cell surface, initiating viral uptake, viral replication, and the production of progeny virus for onward release. Some T‐lymphotropic viruses have developed a strategy to form a stable adhesive junction between an infected cell (effector) and uninfected cell (target), termed a virological synapse (VS). No fusion events take place between the cells; instead, a junction is formed to transfer intact viral particles or genetic material. This process is termed *trans*‐infection (Geijtenbeek et al., 2000) and has been found to be significantly more efficient than infection via cell‐free virus in vitro (Sourisseau, Sol‐Foulon, Porrot, Blanchet, & Schwartz, 2007). HIV‐1 and human T‐lymphotropic virus type 1 (HTLV‐1) are two examples of viruses that trigger the polarisation of cellular machinery and cytoskeleton to form the VS at the cellular interface (Igakura et al., 2003; Jolly, Mitar, & Sattentau, 2007). Transmission of virus from cell to cell in this manner allows the efficient infection of target cells without exposure to the immune system.

In this review, we discuss the methods used by viruses to modulate host cellular machinery and signalling cascades to create a balance between rapid viral replication and establishment of latency. We go on to detail how HIV‐1 and HTLV‐1 use the VS to transfer virus cell to cell and the importance of this in vivo and briefly look at how other viruses may use similar methods of cell‐to‐cell spread.

## 2 IMMUNOLOGICAL SYNAPSE

T‐cell activation is dependent on the formation of the IS; once bound to an APC, the T‐cell is able to detect specific peptide–major histocompatibility complex (pMHC) complexes (Xie, Tato, & Davis, 2013) and respond by polarising receptors and directing membrane trafficking to the site of contact. The two cells form a stable but transient junction via receptor engagement. The synapse allows the secure secretion of cytokines and lytic granules to mount a tailored immune response to pathogens used as a platform for the release of microvesicles to induce activation of signalling pathways (reviewed in Dustin & Choudhuri, 2016) and the extraction of pMHC from APC by T‐cells during trogocytosis (Osborne & Wetzel, 2012). Recognition of APC by T‐cells results in the reorientation of the microtubule‐organising centre (MTOC), Golgi, and endosomal compartments to the contact site along with receptors, coreceptors, and adhesion molecules including TCR, CD4 or CD8, and the integrin's LFA‐1 and intercellular adhesion molecule (ICAM)‐1, respectively. Filamentous actin and actin‐interacting proteins including talin are also found to accumulate at the junction (Dustin & Choudhuri, 2016). Due to the redirection of membrane trafficking to this contact site, the IS becomes a focal point for both exocytosis and endocytosis regulating the transfer of cellular components (Griffiths, Tsun, & Stinchcombe, 2010).

**Figure 1:**
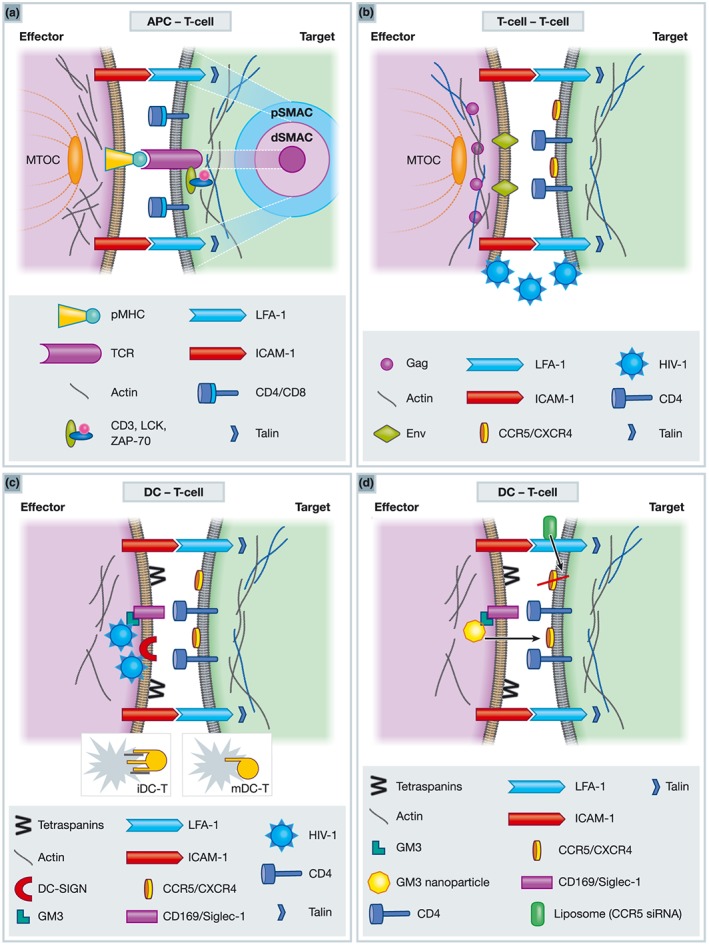
**Schematic representation of immunological (IS) and virological synapses (VS).** (a) Immunological Synapse. In the target cells TCR interacts with pMHC on the effector cells to form a Microcluster (MC) or cSMAc. The pSMAC is formed via interaction of LFA‐1/talin and ICAM1. Actin makes up the dSMAC. CD4/CD8, Lck, Zap‐70 are also recruited to the contact sites of the target cells. (b) Virological synapse between T‐cells. CD4 and CXCR4 expressed on the cell surface on the target cell interacts with viral Env presented at the plasma membrane of the effector cell and LFA‐1 engages ICAM‐1. Virus buds from the effector cell across the synapse and fuses with target T‐cell. (c) Virological synapse between DC and T‐cells. Actin, ICAM‐1 and tetraspanins (CD81, CD63, CD9, and CD82) concentrate on the DC side, whereas CD4, CXCR4/CCR5 and LFA‐1 polarise to the T‐cell contact site. In immature DC, virus is captured via DC‐SIGN and redistributed to the VS. Membrane extensions form between cells through the activation of Cdc42 through Env interaction with DC‐SIGN. In mature DC, GM3 incorporated into the viral particles is targeted to Siglec‐1 (CD169) trafficking virus to the plasma membrane. mDC extend actin membrane sheets around T‐cell (target). (d) Potential drug delivery via the VS using nanoparticle technology. GM3 containing nanoparticles bind to Siglec‐1 and induce VS formation therefore can be used for targeted drug delivery to T‐cells via the VS. Liposomes coated in antibodies against LFA‐1 containing siRNA against CCR5 can reduce HIV viral load.

The TCR engages with the APC major histocompatibility complex (pMHC) triggering IS assembly by forming a TCR/pMHC microcluster (MC) at the contact site. The MC forms the centre of central supramolecular activation complex. The peripheral supramolecular activation cluster (pSMAC) consisting of LFA‐1/ICAM‐1 forms a ring around the central supramolecular activation complex. An additional distal layer surrounds the pSMAC formed by F‐actin associated with CD45. Additional proteins are recruited such as protein tyrosine kinases, Lck, ZAP‐70, and PCKθ, and adaptor protein talin through interaction with LFA‐1 (Figure 1a). TCR engagement with pMHC induces transcriptional upregulation in naïve or resting T‐cells, resulting in T‐cell activation and proliferation (Dustin, Chakraborty, & Shaw, 2010).

### 2.1 Viral manipulation of the immunological synapse

In order to establish an infection within a host, pathogens must adapt to the hostile environment imposed by the immune system by evading detection by surveilling immune cells. Viruses have evolved multiple strategies to hijack and manipulate host cell signalling and machinery to aid their own propagation and persistence. T‐lymphotropic viruses are able to strike a balance between subversion of intracellular signalling and trafficking to impair IS formation and T‐cell activation, while still allowing sufficient T‐cell activation to maintain viral replication. Viruses such as retroviruses, herpesviruses, and paramyxoviruses have developed specific mechanisms to alter TCR‐regulated pathways resulting in inhibition of IS formation and immune detection, while promoting viral replication and release of progeny virus.

#### 2.1.1 HIV‐1 Nef

HIV‐1 and primate simian immunodeficiency viruses (SIV) genomes encode several accessory proteins (*nef*, *vif*, *vpu*, *vpr*, and, in the case of SIV, *vpx*). *Vif*, *vpu*, *vpr*, and *vpx* are linked to the subfamily of ubiquitin ligases and induce the proteosomal degradation of cellular restriction factors, suppressing antiviral activity to allow efficient viral propagation and release. Nef is a key viral protein that is expressed in early infection and determines viral pathogenicity in vivo (Kestler et al., 1991).

Nef has been found to regulate several aspects of the host cell including the intracellular trafficking and downregulation of cellular surface proteins. CD4 (Piguet et al., 1999), CCR5 (Michel, Allespach, Venzke, Fackler, & Keppler, 2005), major histocompatibility complex I and II (Piguet et al., 2000), CD28 (Swigut, Shohdy, & Skowronski, 2001), and SERINCs (Rosa et al., 2015; Usami, Wu, & Gottlinger, 2015) are downregulated, whereas dendritic cell‐specific ICAM grabbing non‐integrin (DC‐SIGN) is upregulated (Sol‐Foulon et al., 2002). However, LFA‐1, ICAM‐1, and ICAM‐2 appear to remain unaffected (Thoulouze et al., 2006). This approach allows HIV‐1 to remain hidden in infected cells by controlling how the cell communicates with the rest of the immune system. An additional advantage to the downmodulation of the expression of viral receptors on the cell surface, such as CD4, helps prevent subsequent reinfection with a closely related viral strain, avoiding “superinfection” of the cell (reviewed in Nethe, Berkhout, & van der Kuyl, 2005).

Nef also targets intracellular signalling and protein trafficking pathways by interacting with various components of the TCR signalling cascade such as Vav‐1 (Fackler, Luo, Geyer, Alberts, & Peterlin, 1999), Erk (Schrager, Der Minassian, & Marsh, 2002), PAK‐2 (Renkema, Manninen, Mann, Harris, & Saksela, 1999), and PKθ (Smith, Krushelnycky, Mochly‐Rosen, & Berg, 1996). The impeded trafficking of TCR receptor from the cell surface leads to retention in recycling endosomes along with Lck (Thoulouze et al., 2006). In conjunction with downregulation of CD4 and CD28 (Brady, Pennington, Miles, & Dzierzak, 1993; Swigut et al., 2001) and Nef's ability to disassociate CD4 from Lck and target it for degradation (Kim, Chang, Kwon, & Rhee, 1999), the targeted attack on TCR signalling reduces clustering at the IS and results in inefficient IS formation.

Nef is also an important regulator of actin cytoskeleton dynamics, through interactions with the GTPase exchange factor Vav1, prompting cytoskeleton rearrangements and activation of c‐Jun N‐terminal kinase/stress‐activated protein kinase cascade (Fackler et al., 1999). Furthermore, Nef interacts with PAK‐2 inhibiting the activity of neural Wiskott–Aldrich syndrome protein and Rac‐1, both regulators of actin polymerisation and T‐cell activation (Haller et al., 2006).

HIV has developed multiple strategies to alter receptor expression, signalling pathways, and cytoskeleton rearrangements resulting in the inefficient formation of the IS. Nonpathogenic SIV is a prime example of how an efficient block to T‐cell activation promotes viral persistence through immune evasion. SIV Nef disrupts the formation of IS between APC and T‐cells through the efficient downregulation of TCR and CD28, therefore blocking T‐cell responses to virally infected cells and avoiding apoptosis. In the case of HIV‐1, some studies suggest Nef is less efficient at preventing IS formation due to a weaker downregulation of TCR and CD28 resulting in increased levels of T‐cell activation and apoptosis (Arhel et al., 2009). Thus, successfully blocking T‐cell activation reduces viral replication permitting prolonged viral production and persistence within the host, whereas failure to actively control T‐cell activation increases replication ultimately resulting in increased pathogenicity and disease progression.

#### 2.1.2 What methods do other viruses use to modulate TCR signalling pathways?

The paramyxovirus human respiratory syncytial virus is a causative agent of respiratory infections worldwide. The nonstructural genes carried by the virus control dendritic cell (DC) maturation and reduce antigen presentation to T‐cells. The N protein is transported to the cell surface of the APC where it interacts in *trans* with TCR molecules. This interaction is believed to inhibit T‐cell activation by downregulating TCR signalling and pMHC clustering resulting in inhibition of IS formation, reviewed by Canedo‐Marroquin et al. (2017).

HTLV‐1 has the ability to control T‐cell activation for its own requirements. The HTLV protein P12^I^ expressed in early infection is capable of inducing T‐cell activation by the activating transcription activator nuclear factor of activated T‐cells and interleukin‐2 production (Albrecht et al., 2002; Ding et al., 2002, 2003; Kim, Ding, Albrecht, Green, & Lairmore, 2003). In addition, viral protein Tax is able to bypass TCR signalling and activate CD28, CD69, and CD5 expression (Chlichlia et al., 1995) promoting T‐cell activation. Conversely, HTLV‐1 reduces TCR cell‐surface expression via downregulation of TCR genes (de Waal Malefyt et al., 1990) and similarly blocks transcription of Lck (Koga et al., 1989), thus controlling IS formation and activation of T‐cells.

Herpes viruses establish lifelong latent infections in host cells. Human herpes virus (HHV) 6 and HHV7 are able to subvert TCR signalling and intracellular trafficking of receptors TCR and CD4 but do not affect levels of Lck (Furukawa, Itoh, Krueger, Streuli, & Saito, 1994; Secchiero et al., 1997). This activity has been attributed to HHV6 U24, which blocks TCR receptors access to recycling endosomes (Sullivan & Coscoy, 2008) and therefore prevents recycling back to the cell surface. Similarly, herpes simplex virus has also developed strategies to remodel TCR signalling to selectively activate TCR pathways. Herpes simplex virus ORF5 is tyrosine phosphorylated upon TCR stimulation and able to interact with SH2 signalling proteins including Lck, which in turn activates TCR signal transduction to promote gene expression and persistent infection (Lee et al., 2004).

Herpes samari (HVS) is an oncogenic simian gamma 2 herpesvirus able to immortalise human T‐lymphocytes. HVS has multiple viral proteins aimed at TCR signalling inhibition. The viral protein Tip (tyrosine kinase‐interacting protein) interacts with Lck and sequesters it along with TCR and LFA‐1 in vesicular compartments (Cho et al., 2004, 2006; Jung et al., 1995; Park et al., 2003). Moreover, Tip interaction with the lysosomal protein p80 results in the degradation of the sequestered Lck (Park et al., 2002), preventing downstream signalling events. Tip is also responsible for the downregulation of CD4 and TCR at the cell surface (Cho et al., 2006; Park et al., 2003), interfering with TCR signalling cascade and IS formation (Cho et al., 2004).

Numerous other viruses have been reported to modulate the TCR signalling pathway to strike a balance between prompting replication and evading detection in the host. To date, these include measles, hepatitis C, vaccinia virus, and Epstein–Barr virus (EBV). For example, DC infected by measles virus have been found to form unstable IS with T‐cells (Shishkova, Harms, Krohne, Avota, & Schneider‐Schaulies, 2007). Through the MV and F/H complex, T‐cell activation is suppressed (Dubois, Lamy, Chemin, Lachaux, & Kaiserlian, 2001), and actin remodelling, T‐cell polarisation, and TCR clustering are inhibited at the IS (Muller et al., 2006; Niewiesk et al., 1999; Shishkova et al., 2007). Similarly, hepatitis C virus (HCV) is reported to downregulate TCR in peripheral T‐lymphocytes (Maki et al., 2003), vaccinia virus VH1 protein can block TCR activation of the interleukin‐2 promoter (Alonso et al., 2002), whereas EBV latent membrane protein LMP2A can bind to Lck, Fyn, and ZAP‐70 downregulating TCR and attenuated TCR signalling (Katzman & Longnecker, 2004), reviewed in Jerome (2008).

Viruses have dedicated multiple specialised proteins to the modulation of TCR signalling showing how integral the IS is in pathogen recognition and killing. For many T‐lymphotropic and APC viruses, it is essential to replicate efficiently and rapidly but go undetected by the host immune system. These two requirements have evolved into controlled modulation of components of the IS and downstream T‐cell activation. Viruses such as those mentioned strike this perfect balance, increasing T‐cell activation at low levels to aid infection and replication while preventing TCR signalling and complete T‐cell activation, to prevent overexpression of viral proteins and apoptosis of the host cell.

## 3 VIROLOGICAL SYNAPSE

The first details of VS were reported in HTLV‐1 transfer between T‐cells (Bangham, 2003) and have subsequently been a central topic of HIV‐1 research in regard to transmission between T‐cells (Jolly, Kashefi, Hollinshead, & Sattentau, 2004) and DC to T‐cell (Arrighi et al., 2004; McDonald et al., 2003; Turville et al., 2004). In fact, HIV‐infected CD4^+^ T‐cells are reported to transmit virus via VS across penile urethral epithelium to macrophages within an in vitro reconstructed mucosal system where a latent infection could be established (Real, Sennepin, Ganor, Schmitt, & Bomsel, 2018). The VS forms between an infected and uninfected cell forming a transient but stable junction to allow the transfer of viral particles. Although VS and IS share a similar structure, the VS has several unique features. The most important of which is that TCR is not found at the VS (Jolly et al., 2004) and the VS lacks the defined MC or distal supramolecular activation cluster of the mature IS (reviewed in Vasiliver‐Shamis, Dustin, & Hioe, 2010).

### 3.1 HIV‐1 virological synapse

#### 3.1.1 T‐cell to T‐cell

The VS is a transient, dynamic structure that forms upon recognition by the HIV glycoprotein gp120 expressed in the effector cell by the surface receptor CD4 on the target T‐cell. The interaction results in the recruitment of the viral Gag polyprotein to the contact site (Jolly et al., 2007) potentially through an interaction with the tumour suppressor adenomatous polyposis coli protein that directly binds HIV‐1 Gag, not only regulating the localization of viral components for HIV‐1 assembly but also enhancing the VS cell‐to‐cell transmission of HIV‐1 (Miyakawa et al., 2017). This in turn triggers the recruitment of the HIV coreceptors CCR5 and CXCR4 along with cellular adhesion molecules ICAM‐1 and LFA‐1 to the VS. Tetraspanins and other surface proteins help form a stable VS to aid viral transmission (Jolly et al., 2004; Jolly & Sattentau, 2007; Starling & Jolly, 2016). The gp120 interaction with CD4 also induces cytoskeleton rearrangements and remodels the actin cytoskeleton, and LFA‐1 induces the polarisation of the MTOC to the interface in the infected T‐cell (Jolly et al., 2007; Starling & Jolly, 2016). Jolly et al. (2004) observed recruitment of CD4, CXCR4, talin, actin, and LFA‐1 on the target cell when cocultured with infected T‐cells. At the same time, the recruitment of viral Env and Gag to the VS in the infected cell with actin concentrated at the intersection was seen (Figure 1b). More recently, a phosphoproteomic approach to analyse mixed populations of infected and uninfected T‐cells identified over 200 cellular proteins involved in viral transfer. Despite the lack of antigen stimulation, TCR signalling was identified as the most activated pathway in both infected and uninfected T‐cells. It was concluded that activation of TCR, Lck, and Zap70 in infected T‐cells mediated by Env was essential for viral transfer to target T‐cells (Len, Starling, Shivkumar, & Jolly, 2017).

The transmission of virus is thought to be instigated by direct budding of virions from infected to uninfected cells in the synaptic cleft and the probable fusion of virions with the plasma membrane of the target cell (Deschambeault et al., 1999; Fais et al., 1995; Pearce‐Pratt, Malamud, & Phillips, 1994; Figure 1b). After the transfer to the recipient cell immature virus has been found to accumulate in endocytic compartments of the target T‐cells leading to maturation of virions and viral membrane fusion, concealing the virus from detection by neutralising antibodies (Dale et al., 2011).

#### 3.1.2 DC to T‐cell

DCs reside in the mucosal tissues and include several subpopulations, including Langerhans (LC) and myeloid DC. DC's main role is to interact with and present pathogen‐derived antigens to the adaptive immune system. These APC cells are perfectly positioned to encounter HIV in early sexual transmission (Zaitseva et al., 1997). Indeed, a subset of vaginal epithelial DCs appear to be important for viral selection during the initial stages of infection as they preferentially replicate CCR5 viruses over CXCR4 and were found to be an important reservoir of infection in vivo (Pena‐Cruz et al., 2018). Exposure to pathogens results in stimulus of DCs and their subsequent maturation and migration to lymphoid tissue where they interact with antigen‐specific T‐cells, leading to T‐cell activation.

HIV‐1 is able to infect DC; however, infection levels are much lower than in CD4^+^ T‐cells. DCs possess several restriction factors to discourage replication, such as dNTP triphosphatase SAMHDI (Berger et al., 2011; Hrecka et al., 2011; Laguette et al., 2011; Ryoo et al., 2014). Interestingly, this is not the case for LC; instead, the cytokine transforming growth factor β signalling pathway is able to potently restrict replication at the transcriptional stage (Czubala et al., 2016). Uptake in immature DC (iDC) is mediated via attachment to CD4, coreceptors CXCR4 or CCR5 and the c‐type lectin DC‐SIGN (Arrighi et al., 2004; Geijtenbeek et al., 2000), whereas LCs use alternative c‐type lectin, langerin (Hu et al., 2004; Turville et al., 2002). After entry into DC, the transfer to target T‐cells can occur by two main routes. Firstly, *cis*‐infection tends to occur in iDC and involves the productive replication and release of progeny virus. The second is *trans‐*infection where DCs capture virions; however, productive infection is absent, and whole intact viral particles are trafficked to T‐cells via a VS (Garcia, Nikolic, & Piguet, 2008; Piguet & Steinman, 2007). *Trans*‐infection is associated with mature DC (mDC). Attachment to DC‐SIGN allows virus to remain infectious for prolonged periods of time in DC (Geijtenbeek et al., 2000) despite the fact that DCs have a highly developed endolysosomal pathway (Blauvelt et al., 1997; Turville et al., 2004). This may at least in part be attributed to the SNARE‐associated protein Snapin downregulating toll‐like receptor 8 signalling in infected DC endosomes (Khatamzas et al., 2017). Instead, virus is sequestered in endosomal‐derived compartments upon maturation (Garcia et al., 2008; Wang et al., 2017). Compartments are found to be rich in tetraspanins such as CD81, CD82, CD9, and CD63 but absent for lysosomal marker LAMP1 (Garcia et al., 2008, Wang, Eng, et al., 2017). In‐depth imaging studies have revealed these to be continuous with the cell membrane (Bennett et al., 2009; Mlcochova, Pelchen‐Matthews, & Marsh, 2013; Nkwe, Pelchen‐Matthews, Burden, Collinson, & Marsh, 2016), at least in the case of macrophages.

Upon contact of the DC with T‐cell, the enrichment of HIV near the cell surface allows formation of VS (McDonald et al., 2003). Engagement of sialoadhesin CD169 (Siglec‐1) expressed on the surface of mDC with the ganglioside GM‐3 contained in the viral membrane triggers relocation to the cell periphery to initiate VS formation (Izquierdo‐Useros et al., 2012; Izquierdo‐Useros et al., 2012; Puryear et al., 2013; Puryear & Gummuluru, 2013; Puryear, Yu, Ramirez, Reinhard, & Gummuluru, 2012). It has been recently reported that the interaction of these molecules alone is enough to initiate VS formation (Yu et al., 2015). In DC, there is an enrichment of tetraspanins, actin, and ICAM‐1 at the contact site, whereas adhesion molecule LFA1 and HIV receptors CD4 CXCR4/CCR5 concentrate at the surface on the T‐cell side (Cavrois, Neidleman, Kreisberg, & Greene, 2007; Felts et al., 2010; Garcia et al., 2005; Geijtenbeek et al., 2000; Turville, Aravantinou, Stossel, Romani, & Robbiani, 2008; Yu, Reuter, & McDonald, 2008; Figure 1c). Disruption of actin remodelling and microtubules with inhibitors has been shown to prevent VS formation highlighting the importance of the role of the actin cytoskeleton in VS formation (Felts et al., 2010; Menager & Littman, 2016; Nikolic et al., 2011).

Imaging of the VS has revealed the presence of extensive filopodial extensions extending from CD4^+^ T‐cells to mDC and evidence for the formation of sheet‐like membrane extensions that extend around T‐cells (Do et al., 2014; Felts et al., 2010; Figure 1c). In iDC, the formation of membrane extensions is induced via the interaction of HIV Env with DC‐SIGN, which in turn activates the GTPase CDC42 (Nikolic et al., 2011). Furthermore, tetraspanin TSPAN7 and dynamin 2 (DNM2) roles in actin nucleation and cortical stabilisation are essential for maintaining viral particles on dendrites (Menager & Littman, 2016). Membrane extensions are thought to allow contact with the uninfected cell and aid the efficient transfer of virus to promote infection.

To date, most studies of viral cell‐to‐cell transfer have been conducted in vitro; therefore, the importance of the VS and the spread of virus in vivo are starting to be addressed. In a recent study, Murooka et al. show HIV‐1‐infected T‐cells contribute to the systemic infection in a humanised mouse model where productively infected T‐cells were visualised migrating to lymph nodes. A subset of cells were observed forming syncytia and adhering to CD4^+^ lymph node cells resulting in the formation of membrane tethers that may facilitate cell‐to‐cell spread (Murooka et al., 2012; Sewald, Motamedi, & Mothes, 2016). It was later shown that murine leukaemia virus and HIV‐1 are captured by CD169/Siglec‐1 expressed on the cell surface of macrophages. The macrophages formed synapses between B‐1 cells that migrate into lymph nodes to continue to spread via VS, showing the importance of CD169 in viral spread (Sewald et al., 2015). In 2016, Law et al. looked at the genetic patterns of HIV‐1 infection and found the cotransmission of two viral genotypes and the microclustering of infected cells formed, harbouring the same genotype within lymphoid tissue. HIV‐1‐infected cells were able to induce the arrest of the interacting CD4^+^ T‐cells through Env‐dependent cell contacts (Law et al., 2016). Understanding cell‐to‐cell spread in various tissue types will be vital to the development of effective antiviral strategies in the future to block viral transmission to target cells.

### 3.2 HTLV‐1 virological synapse

Infection with HTLV‐1 has been implicated in several diseases including adult T‐cell lymphoma and a range of inflammatory diseases. The primary target of HTLV‐1 is CD4^+^ T‐cells; however, there is evidence for infection of a range of immune cells including DC (Knight, Macatonia, Cruickshank, Rudge, & Patterson, 1993; Macatonia, Cruickshank, Rudge, & Knight, 1992), macrophages (Nath, Ruscetti, Petrow‐Sadowski, & Jones, 2003), B‐cells (Koyanagi et al., 1993), and CD8^+^ T‐cells (Hanon et al., 2000). The virus is taken into T‐cells via the receptor GLUT1 (Manel et al., 2003); however, unlike HIV‐1 transmission, HTLV‐1 is dependent entirely on cell‐to‐cell contact. Once infected, viral transmission is initiated via the binding of adhesion molecule ICAM‐1 on the surface of the infected cell and LFA‐1 on the surface of the target cell (Kim, Nair, Fernandez, Mathes, & Lairmore, 2006). This is in addition to the interaction of the viral Tax protein with ICAM‐1 that appears to promote MTOC polarisation to the contact site (Nejmeddine, Barnard, Tanaka, Taylor, & Bangham, 2005). Viral Env glycoprotein, core proteins p19 and p15, and adhesion molecule talin all polarise towards the junction with the virus receptor GLUT‐1 (Takenouchi et al., 2007), which along with neurophilin 1 (Ghez et al., 2006) and heparan sulphate proteoglycans (Pinon et al., 2003) are thought to strengthen the cell‐to‐cell adhesion. Interestingly, the HTLV VS appears to have a more ordered structure than HIV‐1 VS, due to the recruitment of talin that forms a ring‐like structure, similar to the pSMAC of IS (Igakura et al., 2003). It has been reported that the HTLV‐1 protein P8 downregulates TCR signalling (Fukumoto et al., 2007, 2009), increasing cell contact through interaction with LFA‐1 clustering and controlling membrane extensions between T‐cells (Van Prooyen et al., 2010). Additionally, there is evidence for an alternative route of transmission via extracellular biofilms. The biofilm is believed to store virus particles on the cell surface in carbohydrate‐rich matrices consisting of collagen, agrin, tetherin, and galectin, which transfer between cells upon contact (Pais‐Correia et al., 2010).

Transmission of virus via the VS may provide many advantages to viral survival by evading detection by the host immune system and establishing a latent reservoir of infection between immune cells. This mode of viral transmission has potentially important considerations for existing drug therapies. For example, HIV‐1 transmitted cell to cell requires greater concentrations of broadly neutralising antibodies to neutralise virus when compared with cell‐free virus. In a recent study, several broadly neutralising antibodies were found to have a decreased capacity to neutralise virus isolated from HIV‐1 patients in a transfer assay compared with cell‐free virus (Li, Zony, Chen, & Chen, 2017). Moreover, even though virus transferred via VS is still susceptible to antiretroviral treatment, it is thought to be less sensitive to some commonly used antiretrovirals than cell‐free virus (Sigal et al., 2011). This reduction in sensitivity has been attributed to the accumulation of viral particles at the VS reducing the virus's overall susceptibility to treatment (Duncan, Russell, & Sattentau, 2013). Reduced sensitivity to existing treatments could potentially encourage viral immune escape and contribute to viral persistence in patients, which is an important consideration for future vaccine development.

### 3.3 A common route of cell‐to‐cell transmission?

The formation of the VS provides a powerful and effective route for viral transmission for retroviruses such as HIV‐1 and HTLV‐1; however, recent studies are suggesting this maybe a common mode of transmission between immune cells. A good example is the infection of memory B‐cells with EBV, which results in the recruitment of adhesion molecules and the transfer of virus to polarised epithelial cells (Shannon‐Lowe & Rowe, 2011). Recently, Wang et al. demonstrate that the flavivirus, Japanese encephalitis virus, is taken into DC via DC‐SIGN and plays an important role in *trans*‐infection to T‐cells. Imaging showed the transfer of JEV viral particles from DC to T‐cells via cell‐to‐cell contact and formation of VS (Wang et al., 2017). Similarly, Yang et al. demonstrate that SARS coronavirus, which also uses DC‐SIGN as an attachment receptor, is transferred between DC and target cells via a structure similar to the HIV‐1 VS (Yang et al., 2004). As DC‐SIGN has been reported as an attachment receptor for several other viruses including Ebola (Alvarez et al., 2002), dengue (Tassaneetrithep et al., 2003), human cytomegalovirus (Halary et al., 2002), HIV‐2 and SIV (Pohlmann et al., 2001), and HCV (Wang, Feng, Nie, & Zhou, 2004), it seems plausible that many more diverse viruses use similar methods for transmission to permissible cells.

## 4 NANOPARTICLES TO MIMICK VIRUSES: POTENTIAL THERAPEUTIC TARGETS?

In recent years, the use of nanoparticles in vaccine delivery has become a popular area of research. Drugs, vaccines, and even genes can be encapsulated and delivered to target sites within the body using vehicles such as liposomes, nanospheres/capsules, and micelles (Saravanan et al., 2018; Singh, Kruger, Maguire, Govender, & Parboosing, 2017). In addition, the controlled release, targeted delivery to specific cells or tissues, and greater efficacy produce a potent cell‐mediated and humoral response. Advances in vaccine development via ligand delivery or creation of virus‐like particles have led to several promising treatments for a range of viral infections including HIV‐1, HCV, hepatitis B virus, human papillomavirus, and influenza. Several reviews on the topic detail the current advances (Aikins, Bazzill, & Moon, 2017; Singh et al., 2017; Sulczewski, Liszbinski, Romao, & Rodrigues Junior, 2018).

HIV‐1 vaccine development has demonstrated that coating nanoparticles in the p24 antigen of HIV‐1 allows targeted delivery into the dermis, eliciting a strong, HIV‐1‐specific CD4^+^ T‐cell response and B‐cell antibody production (Caucheteux et al., 2016). Exploiting the mechanics of VS formation has led to the development of ganglioside GM3 membrane‐wrapped gold nanoparticles that were found to activate GM3‐CD169 trafficking pathway in mDC. The addition of GM3 to the virus‐like nanoparticles was enough to deliver the conjugate to CD81^+^ compartments that accumulated at the junction between mDC and T‐cells, resembling the structure of a VS (Yu et al., 2015). Another promising approach incorporates the delivery of CCR5 siRNA encapsulated in liposomes coated in antibodies against LFA‐1. Mice challenged with HIV after treatment with the CCR5 liposomes maintained CD4^+^ cell count and a twofold reduction in viral load (Kim et al., 2010). Overall, the targeted delivery to immune cells via LFA‐1 appears as a promising approach at preventing viral spread (Figure 1d).

In respect to the IS, targeted control of the upregulation or downregulation of TCR signalling maybe beneficial in a range of diseases such as autoimmune disease or chronic infections and therefore a viable therapeutic target (reviewed in Jerome, 2008). The use of this next‐generation drug delivery is a very attractive prospect for the targeted delivery of vaccines exploiting the IS and VS to illicit specific immune responses.

## 5 CONCLUSION

The modulation of TCR signalling and VS formation appear to be an effective mechanism to disseminate virus to target cells and remain undetected by the host immune system. Viruses have evolved to manipulate these cellular adhesions to create the VS. Whether this is a specific targeted action or simply exploitation of existing pathways within immune cells remains to be determined. Further study into these structures and the viruses that utilise them will hopefully lead to more specific therapeutic targeting of life‐limiting infection.

## FUNDING INFORMATION

This study has received funding from the Wellcome Trust Seedcorn Fund Grant/Code AC11900001 and the Department of Medicine University of Toronto.

## CONFLICT OF INTEREST

The authors declare no conflicts of interests.
